# MTTE: an innovative strategy for the evaluation of targeted/exome enrichment efficiency

**DOI:** 10.18632/oncotarget.11646

**Published:** 2016-08-27

**Authors:** Katarzyna Klonowska, Luiza Handschuh, Aleksandra Swiercz, Marek Figlerowicz, Piotr Kozlowski

**Affiliations:** ^1^ European Centre for Bioinformatics and Genomics, Institute of Bioorganic Chemistry, Polish Academy of Sciences, 61-704 Poznan, Poland; ^2^ Department of Hematology and Bone Marrow Transplantation, Poznan University of Medical Sciences, 60-569 Poznan, Poland; ^3^ Institute of Computing Science, Poznan University of Technology, 60-965 Poznan, Poland; ^4^ Institute of Technology and Chemical Engineering, Poznan University of Technology, 60-965 Poznan, Poland

**Keywords:** MTTE, exome enrichment, next generation sequencing, MLPA, capillary electrophoresis

## Abstract

Although currently available strategies for the preparation of exome-enriched libraries are well established, a final validation of the libraries in terms of exome enrichment efficiency prior to the sequencing step is of considerable importance. Here, we present a strategy for the evaluation of exome enrichment, i.e., the Multipoint Test for Targeted-enrichment Efficiency (MTTE), PCR-based approach utilizing multiplex ligation-dependent probe amplification with capillary electrophoresis separation. We used MTTE for the analysis of subsequent steps of the Illumina TruSeq Exome Enrichment procedure. The calculated values of enrichment-associated parameters (i.e., relative enrichment, relative clearance, overall clearance, and fold enrichment) and the comparison of MTTE results with the actual enrichment revealed the high reliability of our assay. Additionally, the MTTE assay enabled the determination of the sequence-associated features that may confer bias in the enrichment of different targets. Importantly, the MTTE is low cost method that can be easily adapted to the region of interest important for a particular project. Thus, the MTTE strategy is attractive for post-capture validation in a variety of targeted/exome enrichment NGS projects.

## INTRODUCTION

Recently, next-generation sequencing (NGS) has become the leading method for analyzing the architecture of human genomes. Although the cost of whole genome sequencing (WGS) has decreased significantly in recent years, it still substantially hampers the use of WGS for large-scale studies involving abundant DNA sample sets [1]. However, it has to be noted that resistance to biases in coverage of some genomic regions (e.g., rich in GC nucleotides) and complete coverage of the genome (especially using most recent PCR-free WGS) are among advantages of WGS [2]. Nevertheless, targeted sequencing, based on the capture and enrichment of a restricted part of a genome (i.e., multiple genomic loci of interest), is currently commonly applied to reduce the costs and the amount of data that requires time-consuming analysis [3]. The use of a targeted enrichment sequencing strategy focused on well-characterized coding sequences, i.e., whole exome sequencing (WES), yields informative results that are easier to interpret [4–6]. Targeted/exome sequencing may be favorable especially for applications that require high-coverage of the analyzed regions for identification of low–frequency sequence variants. Such applications include: identification of somatic mutations in cancer genome, identification of mosaic mutations in disease-related genes, identification of mitochondrial DNA heteroplasmy, or identification of sequence variants in mixed DNA samples (e.g., in forensic genetics). Currently, several popular ready-to-use kits for the preparation and sequencing of exome-enriched libraries (mostly from Agilent, Roche NimbleGen, and Illumina) are commercially available. Studies focused on the comparison of their performance revealed that generally all kits are well established and provide results of comparable quality. However, it has to be noted that drawbacks and differences in some aspects of enrichment technologies, e.g., accuracy of variant detection and presence of enrichment biases associated with sequence characteristics, were also identified [1, 7–13]. For example, Meienberg and colleagues have revealed that currently available exome-enrichment platforms cannot efficiently capture all known coding exons and emphasized the need of constant evaluation of the updated platform versions [11].

Due to the revealed differences in the performance of the exome-enrichment platforms and the high costs associated with the downstream sequencing analysis, a quality control for capture performance and exome enrichment efficiency is highly desirable. The introduction of a post-capture validation step preceding the sequencing analysis may prevent the sequencing of unsuccessfully enriched libraries [14–16].

**Figure 1:**
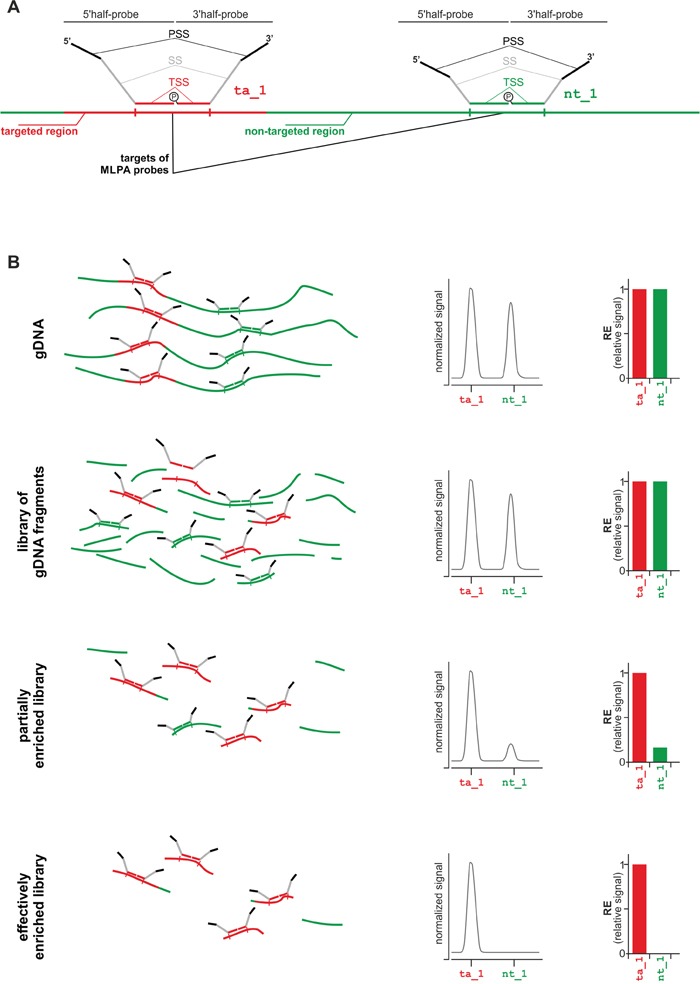
Strategy of MTTE analysis **A.** Schematic representation of targeted region-specific (ta_1; left-hand side) and non-targeted region-specific (nt_1; right-hand side) MLPA probes. Each MLPA probe is composed of two half-probes: a 5′half-probe and a 3′half-probe. Each half-probe is composed of a target-specific sequence (TSS), a primer-specific sequence (PSS), and a stuffer sequence (SS) that allows differentiating MLPA probes by size. More details about the design of MLPA probes may be found in [18, 20]. The first step of the MLPA reaction is hybridization of MLPA probes with the input DNA. Only probes which were correctly hybridized to their targets are subsequently ligated and then amplified with a pair of universal primers. The products of the MLPA reaction are separated in capillary electrophoresis and their relative signals are proportional to the dosage of their targets in the input DNA. **B.** The MTTE analysis of (from the top) gDNA, non-enriched gDNA library (reference), partially enriched library, and effectively enriched library. From the left, schematic representation of the MLPA probes hybridizing to targeted- and non-targeted regions in the input DNA (for simplicity, adapter sequences attached to DNA fragments during library preparation are not indicated in the scheme), electropherograms with signals (peaks) of ta_1 and nt_1 probes, bar-graphs showing relative signals of ta_1 and nt_1 probes.

Here, we developed a new strategy and propose an exome enrichment validation assay, the Multipoint Test for Targeted-enrichment Efficiency (MTTE). MTTE is based on and utilizes the standard well-validated protocol of Multiplex Ligation-dependent Probe Amplification (MLPA) method (Figure 1). The general concept and principle of the MLPA method are discussed in [17–19]. Our assay comprises multiple probes located both in targeted (exome-enriched) and non-targeted genomic regions. In this report, we show that the MTTE is a robust and cost-effective assay that allows the effective and reliable assessment of several enrichment-associated parameters.

## RESULTS

### Design of the MTTE assay

**Figure 2:**
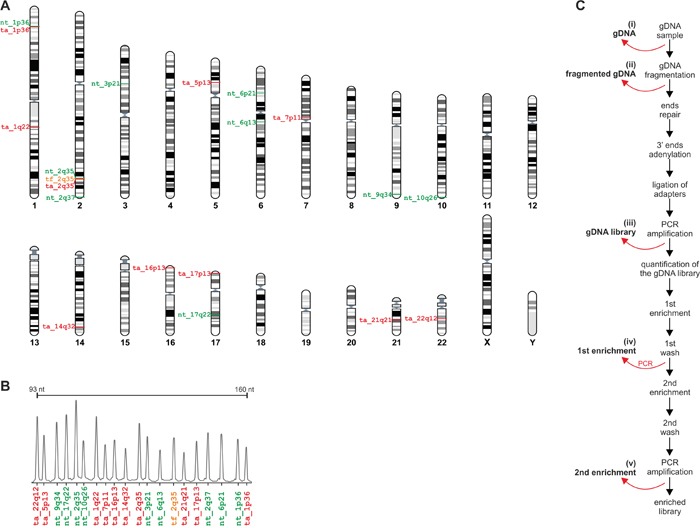
Design of the MTTE assay **A.** The positions of MTTE probes in the human genome. The positions of particular probes are indicated on the chromosome ideograms (left-hand side). IDs of probes located in the targeted regions, in close vicinity (<150) to the targeted regions, and the non-targeted regions are indicated in red, orange and green, respectively. The figure was prepared using the “Ensembl karyotypes” tool available on the Ensembl portal [30]. **B.** Electropherogram showing representative MTTE results from a control gDNA sample from the HapMap panel. Each peak corresponds to the signal of the particular probe indicated below [color coded as in A]. **C.** The workflow of exome enrichment procedure with indicated points at which trace amounts of specimens were obtained for analysis.

Our assay for post-capture exome enrichment validation is composed of 20 MLPA probes, including 10 probes located in targeted genomic regions (mostly exons of protein coding genes and one region overlapping an annotated miRNA sequence), 9 probes located in non-targeted genomic regions (introns and intergenic regions), and one probe located in flank of the targeted regions (49 bp from exon 1 of the *BARD1* gene). The probe located in flank of targeted region was used to test the enrichment of sequences located in close vicinity (≤150bp) to the targeted regions that are enriched together with targeted sequences. The MLPA probe set was designed according to a strategy developed previously in our group, allowing easy design and generation of the assay for the analysis of almost any region of interest [19, 20]. Selected regions were approximately evenly distributed over the genome. To allow the direct comparison of the enrichment efficiency of targeted and non-targeted regions situated in close proximity to each other, in two cases, probes of different types were located in the same gene, i.e., *BARD1* and *ARID1A* (Figure 2A). The designed MLPA probe set was verified to provide robust high quality results in a series of optimization experiments performed using a set of reference gDNA samples (Figure 2B).

### MTTE evaluation of the enrichment-associated parameters

We then used our MTTE assay to analyze the enrichment efficiency in one normal (normal_1) and two acute myeloid leukemia samples (leukemia_1 and leukemia_2). The MTTE analysis of the relative amount of targeted and non-targeted regions was performed during five consecutive steps of the exome enrichment procedure, i.e., (i) untreated gDNA, (ii) fragmented gDNA, (iii) the PCR-amplified gDNA library with ligated adapters, (iv) the PCR-amplified library after the first enrichment, and (v) the PCR-amplified library after the second enrichment (Figure 2C).

**Figure 3:**
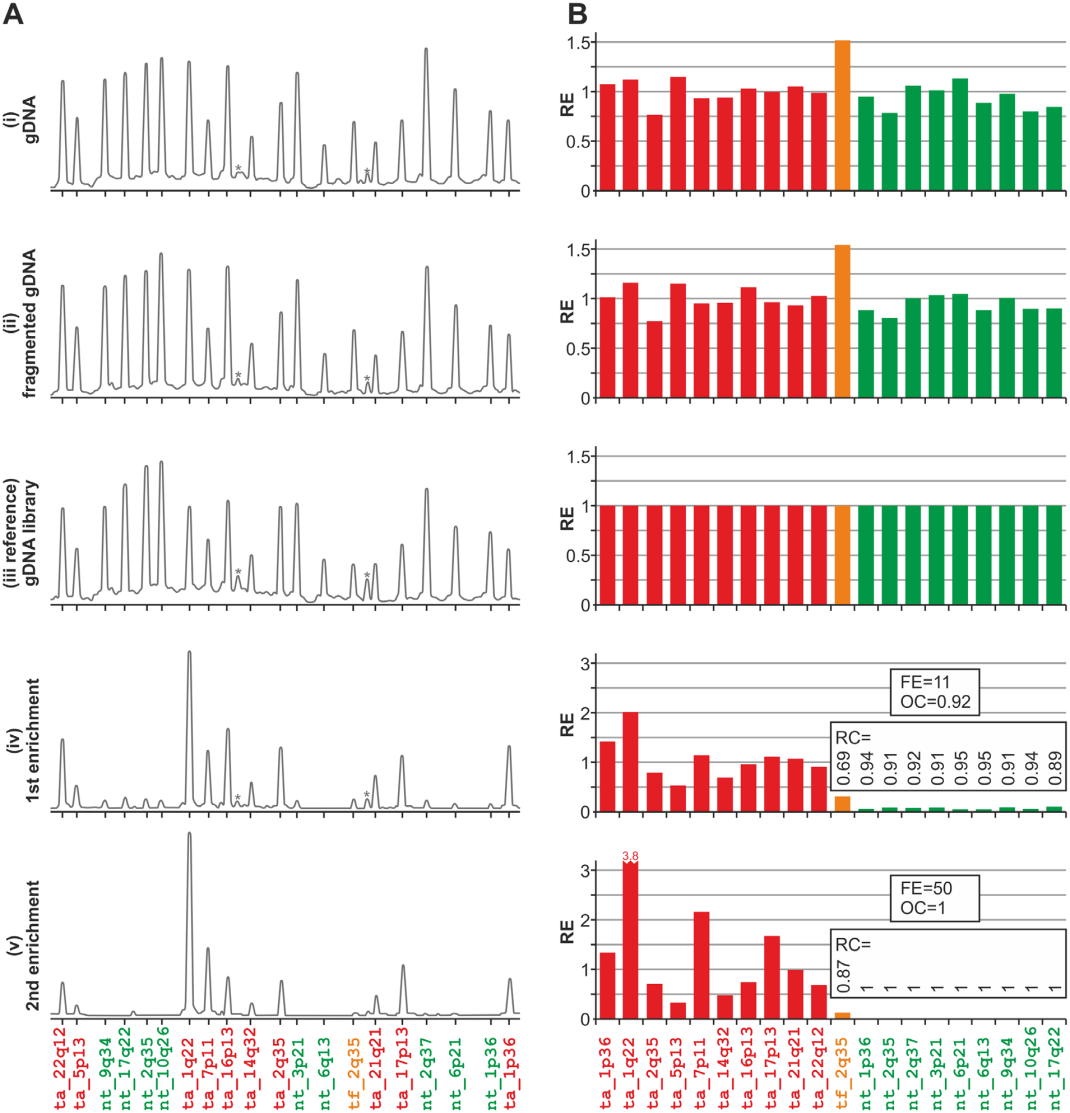
Representative results of the analysis conducted using the in-house designed MTTE assay **A.** The electropherograms of the MLPA results obtained in the analysis of specimens from distinct steps of the exome-enriched library preparation, performed using the leukemia_1 sample. The probe IDs are shown under the electropherograms. Asterisks indicate background signals (unspecific peaks). **B.** The bar plots (corresponding to the electropherograms shown in panel A) representing the relative enrichment (y-axis) of each analyzed region (x-axis). The corresponding RC as well as FE and OC values are indicated on the graphs (steps iv-v).

Representative MTTE results (electropherograms and bar graphs) from leukemia_1 are presented in Figure 3. Bar graphs for all three analyzed samples are shown in Supplementary Figure S1. As shown in Figure 3 and Supplementary Figure S1, DNA fragmentation (ii) and adapter ligation followed by PCR amplification (iii) do not influence the pattern of MLPA signals substantially when compared with the pattern of signals from untreated gDNA samples (i). Therefore, specimens from any of these steps (i-iii) may be considered as a reference for monitoring the exome enrichment. For subsequent analysis, we used the sample from step (iii), which bears structural resemblance to exome-enriched specimens (iv-v), as reference.

As shown in Figure 3 and Supplementary Figure S1, the signal intensities of probes located in the non-targeted regions were drastically reduced after the first enrichment and almost completely disappeared after the second enrichment step.

To quantify the exome enrichment efficiency in steps after the first (iv) and the second (v) enrichment, we calculated the following enrichment-associated parameters (for details, see Materials and Methods): relative enrichment (RE); relative clearance (RC), i.e., 1-RE, calculated for each non-targeted region as well as for target flank region; overall clearance (OC), i.e., average of RC values of non-targeted regions; and fold enrichment (FE), i.e., increase of the fraction of signal intensity of the probes located in targeted regions, weighted by the fraction of the genome covered by the targeted regions. As the regions targeted by the TruSeq Exome Enrichment Kit cover 62 Mb, i.e., 0.02 of the human genome, the theoretical maximum FE is 50. This parameter is comparable to the fold enrichment calculated using the SeqCap qPCR assay from Roche NimbleGen. Analysis of the enrichment-associated parameters revealed the robustness and high reliability of the MTTE assay. The targeted regions were captured with high efficiency. After the first enrichment, the OC reached a value ranging between 0.91 and 0.92, whereas FE was in a range between 10.5 and 11.2. These parameters were significantly improved after the second round of enrichment, with an OC value ranging between 0.98 and 1 and FE reaching a value of up to 50. RC values calculated for individual probes located in the non-targeted regions ranged from 0.83 to 1 and from 0.94 to 1 for the first and the second steps of enrichment, respectively. It should be noted that the clearance of the target flank (the tf_2q35 region), located close to the targeted region (exon 1 of *BARD1*) is much less effective (RC ranging between 0.87 and 0.88 after the second enrichment) than clearance of other non-targeted regions. This result is in agreement with the hypothesis that sequences adjacent to the targeted regions (≤150nt) are also captured by the enrichment procedure. Comparison of the RC values of probes located in the non-targeted regions with their distance to the nearest targeted genomic region further confirmed the positive correlation between the clearance efficiency and increasing distance from the targeted regions (R=0.77, p=0.006). These observations further confirm the specificity of our MTTE assay and correspond to average coverage of sequences surrounding the targeted sequences, which gradually decreases with increasing distance from the targeted sequences and reaches the minimum (∼0) at ∼500 nt upstream and downstream from the targeted regions (Supplementary Figure S2).

### Exome enrichment efficiency: comparison of the MTTE-based evaluation and actual NGS enrichment

For comparison of the MTTE results with actual enrichment, we calculated the fraction of NGS reads mapping to the targeted sequences and the fold enrichment of these values (FE^NGS^), after the first (iv) and second (v) steps of enrichment. As shown in Table 1, the FE calculated based on the MTTE results corresponded well with the FE^NGS^. After the first round of enrichment, both FE and FE^NGS^ reached a value of ∼10, which corresponds to the borderline enrichment value recommended by Roche NimbleGen, manufacturer of the SeqCap qPCR assay. Slightly higher difference between the FE and FE^NGS^ values observed after the second round of enrichment is due to the high disproportion between the fractions of targeted and non-targeted regions (here 1:50). The precision of estimation of fold enrichment decreases when the fold enrichment reaches maximum. As proposed in the SeqCap qPCR validation strategy (SeqCap EZ Library SR User's Guide, v4.2), two-fold differences in the fold enrichment measures should be considered as the same. The higher FE values calculated based on the MTTE results are a consequence of the lower sensitivity of MTTE to detect very low traces of non-targeted sequences, which is due to the limited dynamic range of capillary electrophoresis (very low signals may not be detected and are classified as 0). On the other hand, slightly lower values of FE^NGS^ may be due to the fact that among NGS reads overlapping targeted regions are also those extending to the non-targeted regions. As a result, the calculated fraction of target-associated reads may be higher than the actual fraction of TruSeq targeted regions (>2%).

**Table 1:** Enrichment efficiency (OC, FE, FE^NGS^, and FE^qPCR^) after 1^st^ and 2^nd^ steps of enrichment of the three analyzed samples

	OC		FE		FE^NGS^		FE^qPCR^	
	1st enrichment	2nd enrichment	1st enrichment	2nd enrichment	1st enrichment	2nd enrichment	1st enrichment RN/ratio	2nd enrichment RN/ratio
**Normal_1**	0.91	1	11.2	50	10.9	23.1	8.3/10.5	13.0/34.9
**Leukemia_1**	0.92	1	11.0	50	9.6	23.7	9.5/11.6	14.0/45.1
**Leukemia_2**	0.92	0.98	10.5	32.2	11.3	29.5	12.1/11.9	20.5/32.9

Normal_1, Leukemia_1, and Leukemia_2 – three samples used in the experiment; OC – overall clearance; FE – fold enrichment; FE^NGS^ – fold enrichment of the fraction of NGS reads mapping to the targeted sequences; FE^qPCR^ – fold enrichment calculated based on qPCR analyses; RN – calculated as proposed by the Roche NimbleGen protocol and [16]; ratio – calculated based on ratio of enrichment of targeted and non-targeted regions weighted by proportion of targeted/non-targeted regions in the genome. Note that some differences between FE^qPCR^ (RN) and FE^qPCR^ (ratio) may result from imprecision of measurement of the DNA concentration. Amount of input DNA is assumed to be a normalization factor in FE^qPCR^ (RN) calculation. According to Roche NimbleGen (SeqCap EZ Library SR User's Guide, v4.2), two-fold differences in the FE^qPCR^ (RN) measures should be considered as the same.

### Comparison of qPCR- and MTTE-based evaluation of targeted enrichment

To compare MTTE results with an evaluation of enrichment based on qPCR analysis (proposed before by Roche NimbleGen), we designed and optimized four qPCR assays for targeted regions showing different enrichment efficiency in MTTE experiments. Additionally, we designed three qPCR assays for non-targeted regions, previously analyzed with the use of MTTE. In both cases, the PCR amplicons were designed to maximally overlap target sequences of corresponding MTTE probes. As expected, after the first and second rounds of enrichment threshold cycle (Ct) values decreased for all targeted regions and increased for all non-targeted regions in comparison to the values for the non-enriched library (Supplementary Figure S3). The fold enrichment values calculated based on qPCR results (FE^qPCR^) confirm borderline (∼10) and high quality enrichment (>10) after the first and the second round of enrichment, respectively (Table 1).

Additionally, the direct comparison of the enrichment efficiency of individual probes shows that RE values calculated for the MTTE probes correspond well with FE^qPCR^ values, calculated for the individual qPCR assays (after the first enrichment: R>0.95, p<0.001; after the second enrichment: R>0.85; p<0.01) (Supplementary Figure S3).

### Determination of the structural and sequence-associated features related to bias in enrichment

The results of our MTTE analysis of exome-enriched libraries [steps (iv) and (v)] revealed a considerable difference (up to sixteen-fold) between the RE values of particular targeted probes. This results from the uneven enrichment of different regions. However, it should be noted that the enrichment pattern was recurrent across all analyzed samples (Supplementary Figure S1) and was confirmed in the regions analyzed by qPCR (Supplementary Figure S3). This finding implies that the observed bias in enrichment may result from specific structural and/or sequence-associated features. Thus, we analyzed the potential correlation between the RE values and several features of the targeted regions such as, the length of the targeted region, the occurrence of repetitive elements in the flanking sequences of targeted regions, and the content of different nucleotides in the targeted plus DNA strand (Supplementary Figure S4). The analyses did not indicate the influence of targeted region length and repetitive sequences on the RE of the targeted probes [correlation coefficient (R)∼0]; however, a moderate to high positive correlation was observed between the RE values and the fractions of (i) GC (first enrichment: R=0.59, p=0.06; second enrichment: R=0.40, p=0.23), (iii) purines (first enrichment: R=0.69, p=0.02; second enrichment: R=0.70, p=0.02), and (ii) guanine (G) (first enrichment: R=0.77, p=0.005; second enrichment: R=0.69, p=0.02) nucleotides in the sequence targeted by the TruSeq exome enrichment probes. Consequently, a negative correlation was observed between the RE values and the fraction of thymine (T) (first enrichment: R=0.63, p=0.04; second enrichment: R=0.55, p=0.08) in the targeted regions.

## DISCUSSION

The use of targeted/exome enrichment NGS is becoming increasingly popular. Beside the above mentioned platforms from Agilent, Roche NimbleGen and Illumina, there are also many alternative more tailored approaches, including: MYbaits Target Enrichment Kit (MYcroarray), xGen (IDT), Custom targeted sequencing oligo pools (CustomArray), The RainDance ThunderStorm system (RainDance technologies), Access Array system (Fluidigm), and Quest 5-hmC DNA Enrichment Kit (Zymo Research). Although currently available ready-to-use kits for the preparation of exome-enriched libraries are well established and provide reliable results, a final evaluation of their capture performance and exome enrichment efficiency is of considerable interest due to the high costs associated with the downstream sequencing analysis. Here, we propose an innovative multipoint MTTE strategy that enables the complex evaluation of exome enrichment efficiency using a comprehensively selected and optimized MLPA probe-set. We used the MTTE assay for the calculation of several enrichment-associated parameters that reflect the actual enrichment with high accuracy. The main advantages of our MTTE assay are as follows: (i) MTTE is composed of multiple probes located in targeted sequences of different characteristics. Enrichment of a particular target type (e.g., with higher GC content) may be interpreted in the context of a particular project. (ii) MTTE probes are specific for both targeted and non-targeted regions, therefore allowing the evaluation of enrichment of targeted regions and the evaluation of clearance of non-targeted regions. These measures are complementary and additionally validate each other. (iii) MTTE does not require optimization or generation of standard curves. It takes advantage of a standard protocol (standard reaction conditions, easily accessible reagent set) of MLPA. The standard MLPA setup was validated and successfully used in hundreds of research and clinical studies for the analysis of large mutations in disease-related genes [19, 21–23]. (iv) The MTTE strategy can be easily adapted to the region of interest important for a particular project, e.g., if particular gene is sequenced, target-specific probes may be located in each exon of the gene. (v) The MTTE test is cost-effective (∼5 USD per sample, including cost of the capillary electrophoresis separation, except the starting cost of probes synthesis). (vi) Finally, MTTE may also be used for optimization of enrichment procedures (testing the effect of multiple conditions).

Compared to the commercially available SeqCap qPCR assay, MTTE allows the analysis of more genomic regions (also non-targeted) and utilizes a much simpler setup. MTTE requires the analysis of only two samples, i.e., two MLPA reactions [one reference (not enriched) and one enriched sample] performed in standard conditions, whereas the SeqCap qPCR assay requires 24 reactions, averaging the results (preparation of standard curve) and optimizing the conditions for all fragments to be analyzed. Additionally, our own experience with qPCR analysis indicates that it is much more labor intensive and analysis of each sample requires numerous reactions. The number of required reactions also substantially increases the cost of qPCR analysis (20-50 USD, depending on the used system and number of evaluated regions). However, the MTTE assay is limited by dynamic range of capillary electrophoresis and may miss very low traces of non-targeted probe signals in some samples after enrichment. Nevertheless, this limitation does not affect the reliability of our test to detect poorly enriched libraries (FE<10, recommended by Roche NimbleGen). MTTE also detects libraries with FE=10-25 that do not represent the highest quality of enrichment. It is worth noting that in exome enrichment, FE values of 10 and 25 correspond to 80% and 50% of reads mapping out of the targeted regions, respectively.

Additionally, we took advantage of our MTTE results to determine the potential sequence-associated features that may confer bias in the enrichment of different capture targets. The identified sequence-associated features that increase or decrease the efficiency of targeted enrichment generally overlap with features identified before [e.g., [1, 7, 11, 12]]. For example, we observed a positive correlation between the enrichment efficiency and the fraction of GC in the enriched region (Supplementary Figure S4) as well as between the clearance efficiency and the increasing distance to the nearest targeted region (Supplementary Figure S2). Additionally, our analysis revealed a significant positive correlation between RE values and fraction of purines in targeted sequences that, to our knowledge, has not been reported before. The analysis of the fraction of individual nucleotides showed that the main driver of both purines and GC effect on the enrichment is presence of G in targeted sequences. It may suggest that not only thermodynamic parameters but also sequence composition of either a probe or a targeted sequence may influence enrichment efficiency. It has to be noted, however, that these results should be interpreted carefully due to (i) the non-random selection of probed regions that does not cover the full range of the analyzed parameter (e.g., GC content in a range of 39-60%), and (ii) the low power of our analysis (low number of analyzed regions).

In conclusion, we designed an innovative MTTE strategy for the evaluation of exome-enriched libraries that may be easily adapted to any set of selected targets (e.g., exome, miRNome, methylome (methyl-seq) or panel of genes of interest). The strategy allows not only reliable estimation of general sample enrichment but also allows the evaluation of enrichment of regions of specific characteristics or location that may be of special interest for a project.

## MATERIALS AND METHODS

### Samples

The performance of MLPA probes and the MTTE assay was validated using reference gDNA samples from the HapMap panel purchased from the Coriell Institute for Medical Research [24]. According to the information from the Coriell Institute, all samples were diluted to a working concentration of 50 ng/μl.

The enrichment analysis was performed on 3 samples [one normal (normal_1) and two acute myeloid leukemia (leukemia_1 and leukemia_2) samples] sequenced at the ECBaG (European Centre for Bioinformatics and Genomics) in the framework of other projects.

### Preparation of libraries and sequencing analysis

Three genomic DNA samples (1 μg per sample) were fragmented through sonication [Bioruptor NextGen (Diagenode, Denville, NJ, USA)] at a low power and for 45 cycles (30 s on, 30 s off) and used for the construction of the indexed gDNA libraries using a TruSeq DNA Sample Preparation Kit (Illumina, San Diego, CA, USA), according to the manufacturer's instructions. Whole exome enrichment was performed from 500 ng of each gDNA library using a TruSeq Exome Enrichment Kit (Illumina). For testing of MTTE performance, we compared the efficiency of enrichment after the first and second rounds of enrichment. For this purpose, 10% (3 μl) of the library from the first round of enrichment was pulled out and subsequently treated as the corresponding library after the second step of enrichment (PCR amplification in 10 μl volume). Size distribution of the exome-enriched libraries was validated using the High Sensitivity DNA Assay in a Bioanalyzer 2100 (Agilent Technologies, Santa Clara, CA, USA). The libraries were quantified with a Qubit™ 1.0 Fluorometer (Invitrogen, Life Technologies, Grand Island, NY, USA) and real-time PCR [Rotor-Gene Q (Qiagen, Venlo, The Netherlands)], performed according to the qPCR Quantification Guide (Illumina TruSeq Enrichment Guide) using primers complementary to the adapter sequences and MESA Green qPCR MasterMix (Eurogentec). For sequencing with the Genome Analyzer GAIIx (Illumina), three libraries were combined per lane of a single-read flow-cell in the following way: lane 1 - three libraries before enrichment; lane 2 – three libraries after the first round of enrichment; lane 3 - three libraries after the second round of enrichment. Approximately 9 million 101 bp-long reads were collected for each library. The raw data were submitted to adapter trimming and quality filtering with the FASTX-Toolkit. The remaining reads were mapped to the human reference genome (hg19) using Bowtie 2. To calculate the number of reads mapped to the gene coding and non-coding regions, specialized Linux bash scripts were prepared, taking into account the coordinates of the regions targeted by the TruSeq Exome Enrichment Kit (Illumina).

### MTTE assay and MLPA analysis

MLPA analysis was performed using the in-house designed MTTE assay. The MLPA probes and the probe-set layout were designed and generated according to a previously proposed [19, 20] and well-validated [e.g.,[23, 25–27]] strategy. This strategy exclusively utilizes short oligonucleotide probes that can be easily generated via standard chemical synthesis. Each probe consists of two half-probes of equal size, and the total probe length ranges from 93 to 160 nt. The target sequences for the probes were selected to avoid single nucleotide polymorphisms (SNPs), insertions/deletions, copy number variable regions (CNVRs), segmentally duplicated sequences, repeat elements, and sequences of extremely low or high guanosine-cytosine (GC) content [18, 20]. The designed probes are complementary to the targeted and non-targeted regions, selected based on the genomic coordinates of the regions targeted by the TruSeq Exome Enrichment kit. The sequences and detailed characteristics of all probes are shown in Supplementary Table S1. All probes were selected from MLPA sets designed before and validated in hundreds of samples in our previous projects [19, 20, 23, 25, 26, 28]. The MLPA probes were synthesized by IDT (Skokie, IL, USA). Samples for the MTTE analysis were obtained at several steps during the preparation of exome-enriched libraries. In the MTTE analysis of each sample, we used a 5 μl aliquot of the gDNA/library collected at the following steps of the enrichment procedure: (i) gDNA (50 ng/μl), (ii) 4-fold dilution of 1.25 μl of fragmented gDNA (up to ∼25 ng/μl), (iii) 5-fold dilution of 1 μl of gDNA library, (iv) 5-fold dilution of 1 μl of the exome enriched library after the first enrichment, and (v) 5-fold dilution of 1 μl of the exome enriched library after the second enrichment. The MLPA reactions were run according to the manufacturer's general recommendations (MRC-Holland, Amsterdam, The Netherlands), and as described earlier [17, 19]. The products of the MLPA reactions were diluted 100× in HiDi formamide containing GS Liz600, which was used as a DNA sizing standard, and separated by size using capillary electrophoresis (POP7 polymer; ABI Prism 3130XL apparatus; Applied Biosystems, Carlsbad, CA, USA). The electropherograms were analyzed using GeneMarker software v 2.2.0 (SoftGenetics, State College, PA, USA). The signal intensities (peak heights) were retrieved and transferred to prepared Excel sheets (available upon request). The signal intensity of each probe was divided by the geometric mean of signal intensities of the probes specific for the targeted regions to normalize the values and to minimize run-to-run variation. To calculate RE for each targeted and non-targeted region, the normalized signal intensity of each probe was divided by the corresponding normalized signal of the probe in the reference sample to avoid the biased effect that results from various efficiency of the probes amplification. FE for samples after the first and the second enrichment was calculated according to the following equation: FE=[(TP_E_*TR)/(TP_E_*TR+NP_E_*NR)]/[(TP*TR)/(TP*TR+NP*NR)], where: TP_E_ – the average RE of target probes in the sample after enrichment; TR – the fraction of targeted regions (here 0.02); NP_E_ – the average RE of non-target probes in the sample after enrichment; NR – the fraction of non-targeted regions (here 0.98); TP – the average RE of target probes in the reference sample, which equals 1; and NP – the average RE of non-target probes in the reference sample, which equals 1.

### qPCR analyses

qPCR analyses were performed with the use of LightCycler 480 system with probes from the Universal Probe Library (UPL) (Roche), following the protocol proposed by the manufacturers. qPCR analyses were conducted for all three samples (i.e., normal_1, leukemia_1 and leukemia_2) before enrichment and after the 1st and 2nd steps of enrichment, with the use of four qPCR assays for targeted regions and three qPCR assays for non-targeted regions. The concentration of all samples on each step was measured with the use of the Qubit™ 1.0 Fluorometer (Invitrogen, Life Technologies, Grand Island, NY, USA). For the analyses of leukemia_1, leukemia_2, and normal_1 samples, 1 ng of input DNA from each step of enrichment were used. The sequences of the primers and numbers of probes applied within the individual assays were as follows: ta_1p36 assay – Fwd: 5′-AATAGGGCCTGAGGGAAAC-3′, Rev: 5′-GTAGCGGCTAGGAGAATACAT-3′, probe #57; ta_5p13 assay – Fwd: 5′-ACAGGCCTC TTGGTCTTGT-3′, Rev: 5′-TGTTGCGAAGCTCT TTGGT-3′, probe #17; ta_7p11 assay – Fwd: 5′-GCCAAAAGTGTGATCCAAG-3′, Rev: 5′-GTTTCT GGCAGTTCTCCTC-3′, probe #3; ta_21q21 assay – Fwd: 5′-GCATTAACAGTGTATGATGCC-3′, Rev: 5′-TTATCCAGCAGGGTGACTC-3′, probe #88; nt_2q37 assay – Fwd: 5′- CCCCAAAAAAATCCTCAGA-3′, Rev: 5′- TGGGCTGAAGTTGCTGTAG-3′, probe #60; nt_9q34 assay – Fwd: 5′-ATACTGAGAGGGAAACAGCAG-3′, Rev: 5′-CATAAGCTCCACTTACTGGC-3′, probe #41; nt_17q22 assay – Fwd: 5′- AAAGTC CTGACTCCCTCACT-3′, Rev: 5′- AGAAGTGG GACCAGTGTCT-3′, probe #49. qPCR assays were run for 45 cycles and if particular assay was amplified in or after 36 cycle, we assumed that the Ct value equals 36. For each assay PCR efficiencies (E) were measured based on a standard curve analysis. Fold enrichment (FE^qPCR^) was calculated as E^ΔCt^, where ΔCt is the difference between Ct values of a non-enriched library (reference) and an enriched library (either after the 1st or 2nd enrichment), as proposed by Roche NimbleGen and [16].

### Statistical analysis

All statistical analyses were performed using GraphPad QuickCalcs [29]. All statistical tests were two-sided and p-values less than 0.05 were considered statistically significant.

## SUPPLEMENTARY MATERIAL FIGURES AND TABLE




