# Supplementary material for: MTTE: an innovative strategy for the evaluation of targeted/exome enrichment efficiency
Source: Oncotarget. 2016 Aug 27;7(41):67266–76. doi: 10.18632/oncotarget.11646 (PMC5341873; doi:10.18632/oncotarget.11646)
Supplement: Supplementary file 1 [file oncotarget-07-67266-s001.pdf]

MTTE: an innovative strategy for the evaluation of targeted/exome enrichment efficiency

SUPPLEMENTARY FIGURES AND TABLE

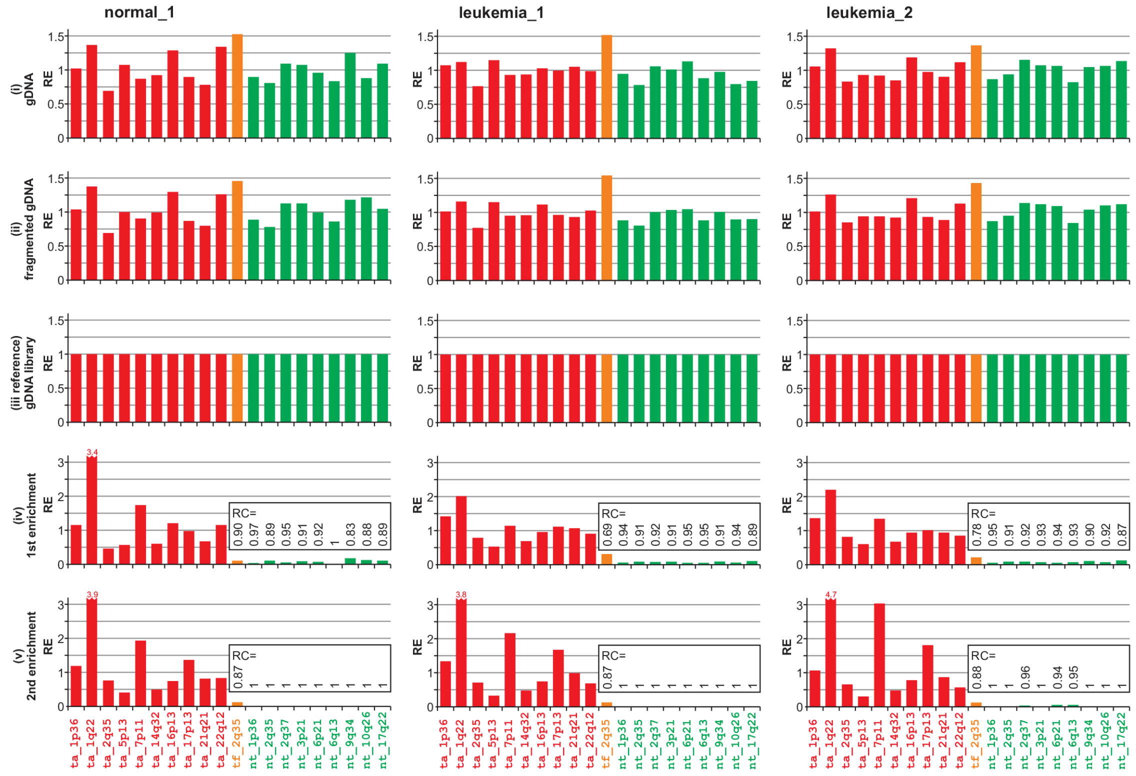

Supplementary Figure S1: Results of the analysis conducted using the in-house designed MTTE assay. The bar graphs represent the relative enrichment (y-axis) of each analyzed region (x-axis), obtained in the analysis of specimens from steps (i-v) of exome enrichment of (from the left) normal_1, leukemia_1, and leukemia_2 samples. The corresponding RC values are indicated on the graphs (steps iv-v).

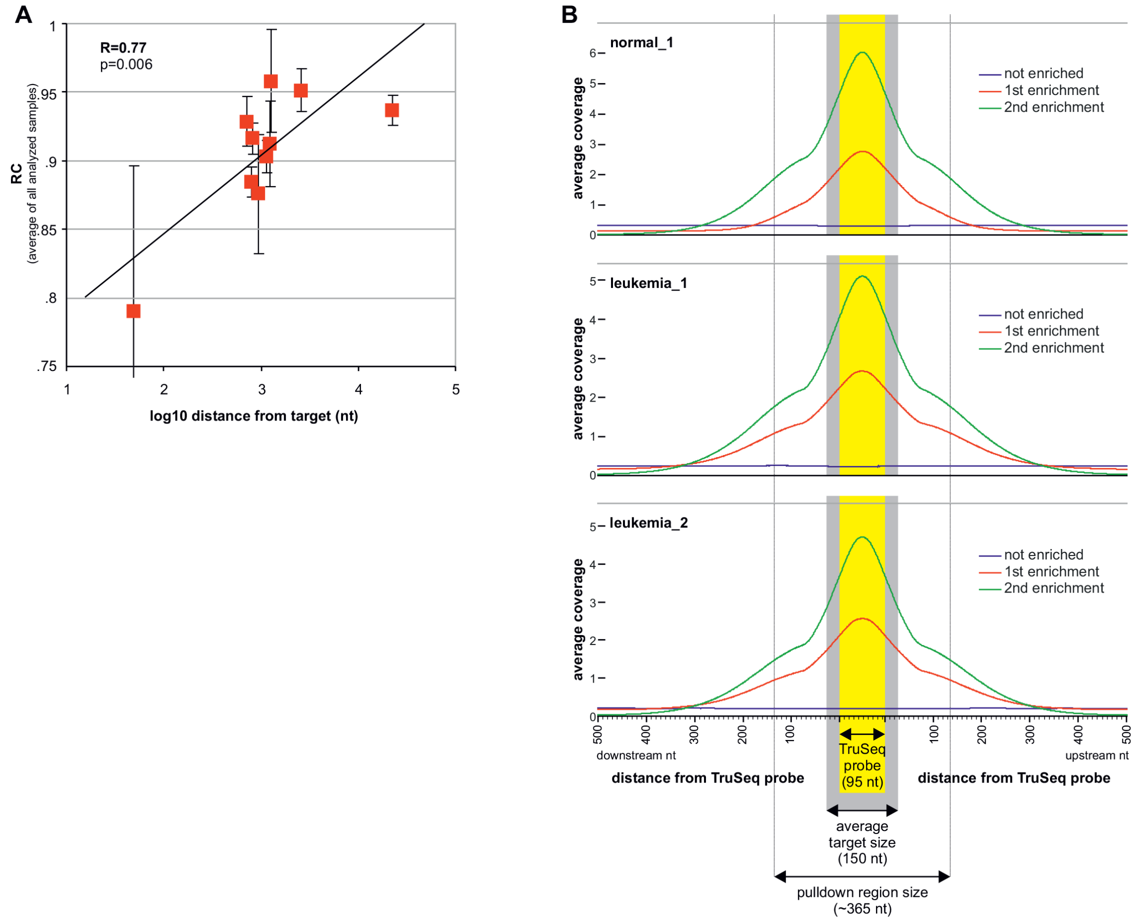

Supplementary Figure S2: Average coverage of the sequences surrounding the targeted sequences. A. The relationship between the clearance efficiency calculated based on the MTTE results and increasing distance from the targeted regions (log10 values). Average (red mark) and standard deviation (SD, error bars) of RC in all analyzed samples (n=3) are indicated. Correlation coefficient (R), p-value for correlation, and linear correlation trend line are shown on the graph. **B.** Plots illustrating an average NGS coverage in relation to the position of the TruSeq exome enrichment probe. For the analysis, we selected 77000 probes that are distant to each other by at least 1000 nucleotides. A targeted region and a pulldown region are indicated on the graph. The pulldown region represents the sequence that may be captured from the library assuming the average size of the library fragments (inserts) to be 230 bp (see technical notes regarding the TruSeq Exome Enrichment Kit).

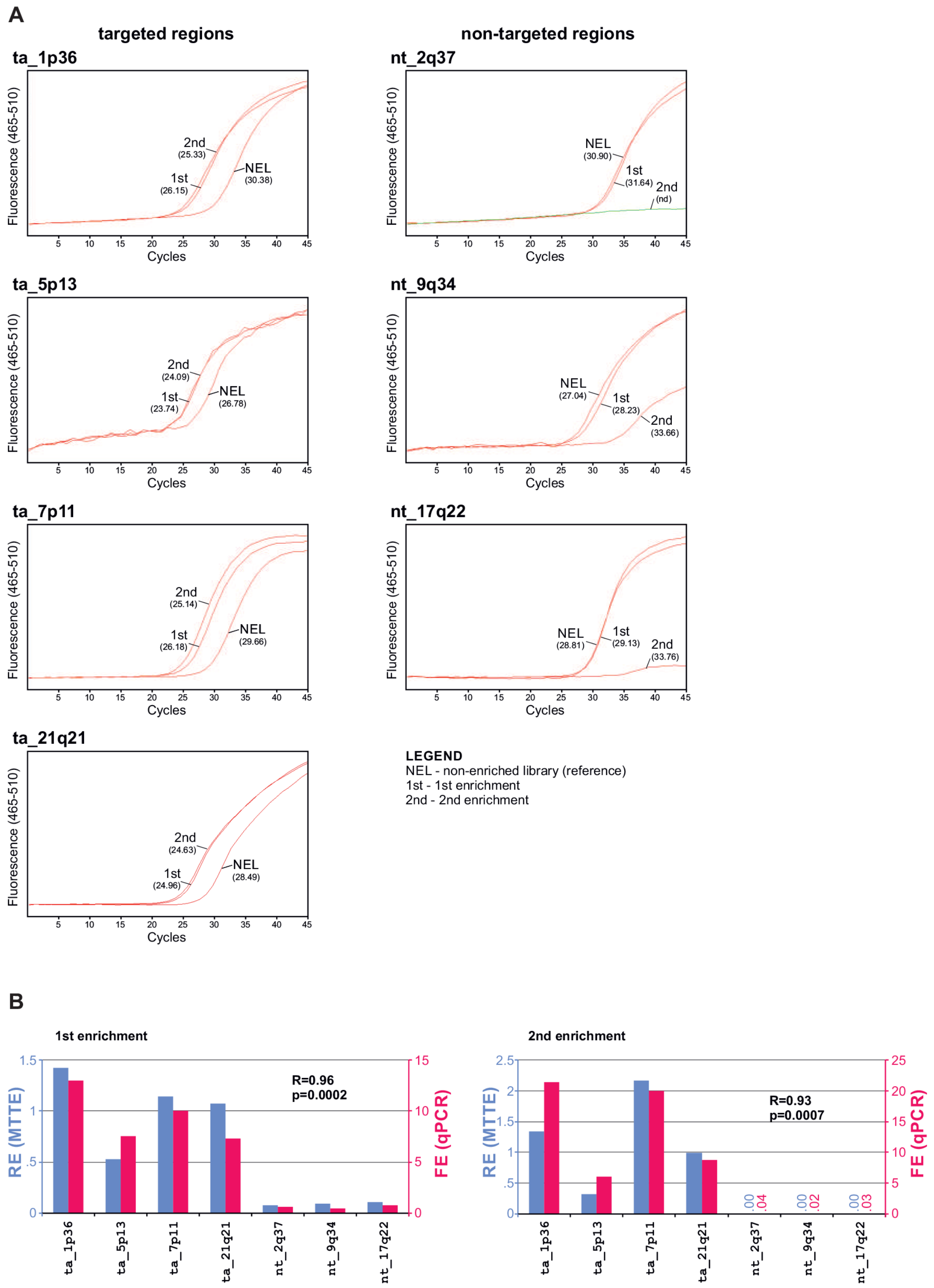

Supplementary Figure S3: Evaluation of the enrichment efficiency with the use of qPCR analysis. A. Representative results of qPCR assays designed for the selected targeted (left-hand side) and non-targeted (right-hand side) regions. The curves representing non-enriched library and libraries after the first and second round of enrichments are indicated on the graphs (corresponding Ct values are indicated in brackets). **B.** Comparison of the RE and FE values (calculated based on MTTE and qPCR results, respectively) corresponding to the selected targeted and non-targeted regions analyzed by both MTTE and q PCR. The figure illustrates representative results obtained for the leukemia_1 sample. The results of the other analyzed samples are very similar.

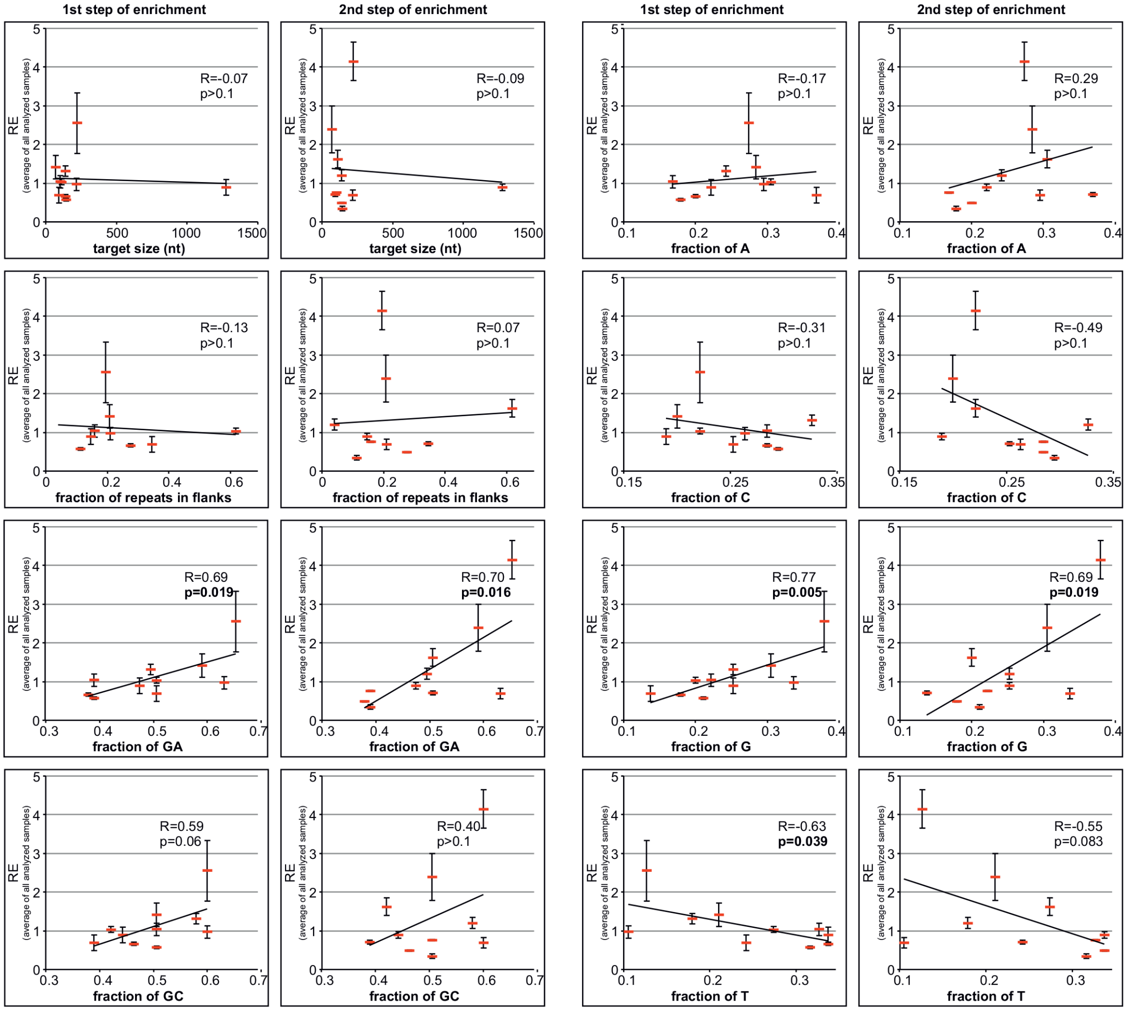

Supplementary Figure S4: Correlation of RE values (y-axis) of targeted probes with potential enrichment-affecting factors (x-axis). Average (red mark) and SD (error bars) of RE in all analyzed samples (n=3) are indicated.R, p-value for correlation, and linear correlation trend line are shown on the graph. Graphs in the left and right columns show the results after the first and second rounds of enrichment, respectively. For the correlation analysis involving fraction of repetitive elements, we took into account a region of 2.5 kb upstream and downstream of the center of TruSeq exome enrichment probes.

Supplementary Table S1: Detailed characteristics and sequences of the probes included in the MTTE assay

See Supplementary File 1
